# Predictive Studies Suggest that the Risk for the Selection of Antibiotic Resistance by Biocides Is Likely Low in *Stenotrophomonas maltophilia*

**DOI:** 10.1371/journal.pone.0132816

**Published:** 2015-07-22

**Authors:** María Blanca Sánchez, Francesca Decorosi, Carlo Viti, Marco Rinaldo Oggioni, José Luis Martínez, Alvaro Hernández

**Affiliations:** 1 Departamento de Biotecnología Microbiana, Centro Nacional de Biotecnología (CSIC), Darwin 3, Cantoblanco, 28049-Madrid, Spain; 2 Dipartimento di Scienze delle Produzioni Agroalimentari e dell’Ambiente, Università degli Studi di Firenze, Florence, Italy; 3 Department of Genetics, University of Leicester, Leicester, LE1 7RH, United Kingdom; University Roma Tre, ITALY

## Abstract

Biocides are used without restriction for several purposes. As a consequence, large amounts of biocides are released without any control in the environment, a situation that can challenge the microbial population dynamics, including selection of antibiotic resistant bacteria. Previous work has shown that triclosan selects *Stenotrophomonas maltophilia* antibiotic resistant mutants overexpressing the efflux pump SmeDEF and induces expression of this pump triggering transient low-level resistance. In the present work we analyze if two other common biocides, benzalkonium chloride and hexachlorophene, trigger antibiotic resistance in *S*. *maltophilia*. Bioinformatic and biochemical methods showed that benzalkonium chloride and hexachlorophene bind the repressor of *smeDEF*, SmeT. Only benzalkonium chloride triggers expression of *smeD* and its effect in transient antibiotic resistance is minor. None of the hexachlorophene-selected mutants was antibiotic resistant. Two benzalkonium chloride resistant mutants presented reduced susceptibility to antibiotics and were impaired in growth. Metabolic profiling showed they were more proficient than their parental strain in the use of some dipeptides. We can then conclude that although bioinformatic predictions and biochemical studies suggest that both hexachlorophene and benzalkonium chloride should induce *smeDEF* expression leading to transient *S*. *maltophilia* resistance to antibiotics, phenotypic assays showed this not to be true. The facts that hexachlorophene resistant mutants are not antibiotic resistant and that the benzalkonium chloride resistant mutants presenting altered susceptibility to antibiotics were impaired in growth suggests that the risk for the selection (and fixation) of *S*. *maltophilia* antibiotic resistant mutants by these biocides is likely low, at least in the absence of constant selection pressure.

## Introduction

Biocides constitute a group of antimicrobials used in several cleaning and general disinfection practices [1–3]. They are commonly used in medicine, agriculture, forestry, industry and even as forming part of very common household and personal care compounds, including toothpastes, cosmetics, soaps and textiles among others. The mechanisms of action of biocides have not been studied in detail and in most cases it is assumed that they present multiple targets [4]. Nevertheless some information on specific targets is available. In this regard, it has been described that triclosan inhibits the enoyl-acyl carrier protein reductase enzyme (FabI) [5,6]. Quaternary ammonium compounds as benzalkonium chloride bind to the phospholipids and proteins of the cell membranes thereby impairing permeability [7], and likely present other intracellular targets, including the DNA [8]. Finally it has been described that hexachlorophene may inhibit respiration and produce bacterial lysis [9,10], but its mechanism of action is not fully understood [11].

Although biocides are of wide use for the aforementioned disinfection procedures, they are not regularly used for treating infections and consequently the regulation rules associated to their utilization are different than those of antibiotics. Furthermore, their wide use has led to the release of large amounts of biocides into natural ecosystems. Consequently, different concerns on the use of such compounds have been raised. In occasions such concerns refer to a potential direct effect of biocides on human health. In other cases, the concerns refer to the effect of these bactericidal compounds on the population dynamics of bacterial pathogens, which also may impact infectious diseases and consequently human health. This possibility has been mainly explored in the case of antibiotic resistance. Indeed, in the last few decades, some works have shown that *in vitro* selected biocide resistant mutants presented also resistance to antibiotics [12–16]. In most cases, resistance was due to the overexpression of multidrug (MDR) efflux pumps capable to extrude both biocides and antibiotics [15–21]. As the consequence of these results a concern was raised on the possible effect that the wide and non-restricted use of biocides for several purposes, as well as their release in waste-water treatment plants and in natural ecosystems may have on the selection of antibiotic resistant microorganisms [1,22–24]. This selection may occur at different environmental compartments, including human linked environments as hospital or homes, but also natural ecosystems, which constitute the final fate of these compounds. Because of this, we have used as a model organism for studying the effect of biocides on the selection of antibiotic resistance the opportunistic pathogen *Stenotrophomonas maltophilia*. This Gram-negative bacterium is a regular colonizer of the roots of the plants [25], but in addition is an increasing cause of infections at hospitals [26,27]. As a consequence of this ecological versatility, *S*. *maltophilia* might acquire resistance in any of the ecosystems (hospitals, houses or natural environments) in which presence of biocides might be expected.

Commonly used biocides belong to different families, being triclosan, benzalkonium chloride and hexachlorophene among the most widely used. We have previously shown that triclosan can select mutants of *S*. *maltophilia* that display a reduced susceptibility to antibiotics [15]. The mechanism of resistance was determined to be the overexpression of the MDR efflux pump SmeDEF. Further work showed that, in addition of selecting mutants when present at inhibitory concentrations [15], subinhibitory triclosan concentrations induce the expression of the *smeDEF* efflux pump [28] due to binding of the biocide to SmeT, the local transcriptional repressor of the pump [29–31]. The conformational changes in SmeT upon triclosan binding render the protein unable to bind the *smeDEF* promoter leading to the transient overexpression of *smeDEF* while triclosan is present [28].

In the present work, we explore the effect that hexachlorophene and benzalkonium chloride may have on transient induction of antibiotic resistance of *S*. *maltophilia*, mediated by SmeDEF overexpression, as well as on the selection of antibiotic resistant mutants upon exposure to these biocides.

## Experimental Procedures

### Bacterial strains and growth conditions

The strain *S*. *maltophilia* D457, its isogenic mutant D457R [32], which overexpresses SmeDEF, and the spontaneous mutants described in the present work were grown in LB medium [33] at 37°C, unless indicated otherwise.

### *In silico* prediction of the interaction of Smet with biocides

The interaction of benzalkonium chloride and hexachlorophene with SmeT was predicted using the AutoDock4 software [34], considering flexibility of the ligands but not for the protein, and restricting the docking area to the protein’s binding pocket. The obtained results were analyzed using AutodockTools 1.5.4, and the predicted protein-biocide complexes were visualized using PyMol [35].

### DNA labeling and electrophoretic mobility shift assay (EMSA) in the presence of biocides

The probe for the EMSA was a 158-bp double stranded [γ-^32^P]dATP 5’end-labeled DNA that contains the SmeT operator site, obtained as described in [29]. The labeled probe (2 nM) was incubated in binding buffer (10 mM Tris-HCl, 50 mM KCl, 10 mM MgCl_2_, 1 mM EDTA, pH 7.2, 50 μg/ml bovine serum albumin, 1 mM dithiothreitol, 5% (v/v) glycerol, and 100 μg/ml poly dI:dC) with purified 0.2 μM SmeT for 20 min at room temperature. Different concentrations of hexachlorophene and benzalkonium chloride (0.1 mM and 0.2 mM of each) were added to the mixture and incubated for 15 min. Protein/DNA complexes were separated on a 6% non denaturing polyacrylamide gel (37.5:1 acrylamide:bisacrylamide). Electrophoresis was performed in 89 mM Tris borate, 2 mM EDTA buffer for 90 min at 100 V and gels were dried before autoradiography.

### Mutants selection and determination of mutation frequencies

Each tube containing LB was inoculated with one isolated colony and incubated overnight. 100 μl of different dilutions (10^0^ to 10^−7^) of the overnight cultures was seeded onto Mueller Hinton agar plates without biocide, or containing the biocides; benzalkonium chloride (128 μg/ml) or hexachlorophene (16 μg/ml). After 48 h at 37°C colonies were counted and mutation frequencies estimated as the ratio between colonies growing in the presence or in the absence of biocides. Mutants capable to grow at 128 μg/ml of benzalkonium chloride or 16 μg/ml of hexachlorophene were chosen for further characterization.

### Real time RT-PCR

Real time RT-PCR was mainly done using the conditions and primers described in [36]. 15 μl of an *S*. *maltophilia* D457 overnight culture were used to inoculate 15 ml of LB containing sub-inhibitory concentrations of hexachlorophene (1.5 μg/ml) or benzalkonium chloride (3.5 μg/ml). When OD_600_ ≈ 1.0, cells were spun down at 6,000 x g for 10 minutes at 4°C, and immediately frozen on dry ice, and stored at -80°C. Total RNA was extracted from cell pellets using RNeasy Mini Kit (QIAGEN) and to further eliminate any remaining DNA, TURBO DNA-free (Ambion) was used. RNA integrity was verified on a 1% agarose gel and absence of DNA was verified by real time PCR using *GyrA-RT*.*fw* and *GyrA-RT*.*rv* primers [36]. cDNA was obtained from 1μg RNA using High Capacity cDNA Reverse Transcription Kit (AB Applied Biosystems). RT-PCR was performed according to Morales *et al* [37] using Power SYBR Green Kit (Applied Biosystems) as indicated by the manufacturer. Briefly, a first denaturation step at 95°C for 10 min followed by 40-cycles (95°C for 15 s, 60°C for 1 min) for amplification and quantification. *GyrA-RT*.*fw* and *GyrA-RT*.*rv* were used to amplify the reference gene *gyrA* as described [36]. Differences in the relative amount of the analyzed mRNAs gene were determined according to the 2^-ΔΔCt^ method [38]. In all cases, the mean values of relative mRNA expression obtained in three independent triplicate experiments were considered.

### Whole genome sequencing and determination of mutations

Whole genome sequencing of two selected mutants and of the wild-type strain D457 was performed as previously described [39] at the *Unidad de Genómica Antonia Martín Gallardo*, *Parque Científico de Madrid*, Spain, following a single read 1x75 protocol and using an Illumina GAIIx apparatus.

The mutations were identified by the Computational Genomics Service of the CNB. Reads were aligned against the *S*. *maltophilia* D457 [40] genome by using the Burrows-Wheeler Alignment tool [41], and single nucleotide polymorphisms (SNPs) were detected using samtools/mpileup/bcftools/vcfutils [42]. Final SNPs candidates were selected by removing those in which the SNP was present in <50% of the reads. Gene annotations and coordinates were obtained from NCBI (accession code HE798556).[39] In all cases the presence of the SNP was confirmed by amplifying and sequencing the DNA region containing the mutation. To that purpose the corresponding genes were amplified using genomic DNA as template, 1 μM of each primer and the enzyme Expand Long Template (Roche). The primers were 0261F (5´ cggaattcttcatgcgtatccttctgg 3´, *Eco*RI site underlined) and 0261R (5´cccaagcttggctcagccctcgttgcg 3´, *Hin*dIII site underlined) for Bz6 strain, and 2876F (5´cggaattcccaatgaaatccaccgcg 3´, *Eco*RI site underlined) and 2876R (5´ cccaagcttgaatcagggattgctggc 3´, *Hin*dIII site underlined) for Bz4 strain. The reaction included one denaturation step at 94°C for 5 min followed by 35 amplification cycles of 94°C for 30 s, 50°C for 45 s and 68°C for 1 min, with a final extension step of 68°C for 7 min. The PCR products were cloned in pGEM-T and sequenced with universal primers by Macrogen (http://dna.macrogen.com/eng/).

### Cloning of the mutant and wild-type alleles of *phoP* and *SMD_2876* in *S*. *maltophilia*

The wild type and mutant alleles of *phoP* and SMD_2876 were amplified by PCR and cloned in pGEM-T as described above. Each gene was excised from pGEM-T using the enzymes *Eco*RI and *Hin*dIII and cloned into pVLT31 [43] using the same enzymes. Four different plasmids were obtained, pBS37 (pVLT31 with the mutant *phoP* allele from Bz6), pBS38 (pVLT31 with the wild-type *phoP* allele from D457), pBS41 (pVLT31 with the mutant SMD_2876 allele from Bz4) and pBS42 (pVLT31 with the wild-type SMD_2876 allele from D457). Plasmids were introduced into D457 (pBS37 and pBS41), Bz4 (pVLT31 and pBS42) and Bz6 (pVLT31 and pBS38) by tripartite conjugation [44]. The exconjugants were selected in LB agar plates with 10 μg/ml tetracycline and 20 μg/ml imipenem.

### Antibiotic susceptibility assays

Minimal inhibitory concentration (MIC) was determined by antibiotic strips (Liofilchem) in Mueller Hinton II containing 0.5 mM IPTG medium. For further testing small changes in susceptibility, spot tests were performed in plates of Mueller Hinton II agar plus 0.5 mM IPTG without antibiotic (control), 2 μg/ml ciprofloxacin or 2 μg/ml ceftazidime. 5 μl of serial dilutions (10^0^, 10^−1^, 10^−2^ an 10^−3^) of overnight cultures were spotted and the growth analyzed after incubation at 37°C for 24h.

### Competition assays

Three different competition assays were performed. A flask with 25ml of LB were co-inoculated with overnight cultures of *S*. *maltophilia* D457, Bz4 and Bz6, a) with the same number of colony forming units (cfu) (50%) of D457 and Bz4 or Bz6; b) 99% of D457 cells and 1% of mutant cells (Bz4 or Bz6); c) 99% of D457 cells and 1% of mutant cells (Bz4 or Bz6) with 0.5 μg/ml ciprofloxacin (1/4 CMI of D457). The co-cultures were grown during four days with daily dilution 1:1000 in new medium without or with antibiotic. Every 24 h sequential dilutions of the co-cultures were spread in LB and LB plus 4 μg/ml ciprofloxacin. For each assay, the total number of cfus, which include both the wild type and the mutant strain, were recorded after 24 h at 37°C in LB plates, and the cfus of the mutant strain were recorded after 48 h at 37°C in LB plates containing 4 μg/ml ciprofloxacin. Data were represented as log of mutant cfu divided by D457 cfu. The assays were performed in duplicate

### Metabolic profiling

The *S*. *maltophilia* strains D457, D457R, Bz4 and Bz6 were tested on PM plates PM1-2 (carbon sources), PM4 (phosphorus, sulfur sources), PM5 (growth factors) and PM3,6,7,8 (nitrogen sources). The complete list of the compound assayed can be obtained at http://www.biolog.com/pdf/PM1-PM10.pdf. PM uses tetrazolium violet reduction as a reporter of active metabolism [45]. The reduction of the dye causes the formation of a purple color that is recorded by a CCD camera every 15 min and provides quantitative and kinetic information about the response of the cells in the PM plates [45]. The strains were grown overnight at 30°C on BUG and then cells were picked up with a sterile cotton swab and suspended in 15 ml inoculation fluid IF-0 1x (Biolog). Cell density was adjusted to 42% transmittance (T) on a Biolog turbidimeter.

PM1 and PM2 were inoculated, 100 microliter each well, with a fluid obtained diluting the cellular suspension (42% T) 6 times in 1x IF-0 (Biolog) added 1x Dye Mix A (Biolog). PM3, PM4 (rows A,B,C,D), PM5, PM6, PM7, PM8 were inoculated, 100 microliter each well, with a fluid obtained diluting the cellular suspension (42% T) 6 times in 1x IF-0 (Biolog) added with 20 mM sodium succinate, 2 μM ferric citrate, 50 μM L-methionine and 1x Dye Mix A (Biolog). PM4 (rows F,G,H) was inoculated, 100 microliter each well, with a fluid obtained diluting the cellular suspension (42% T) 6 times in 1x IF-0 (Biolog) added with 20 mM sodium succinate, 2 μM ferric citrate and 1x Dye Mix A (Biolog). All the PM plates were incubated at 37°C in an Omnilog Reader (Biolog). PM experiments were performed in duplicate.

PM plates were monitored by the Omnilog Reader automatically every 15 min for color changes in the wells. Readings were recorded for 48 h, and data were analyzed with Omnilog-PM software (release OM_PM_109M) (Biolog), which generated a time-course curve for tetrazolium color formation. The data from the Omnilog-PM software were filtered, using area as a parameter. For each mutant the parameter ΔA was calculated for each well of PM panels. ΔA was defined as ΔA = A_MT_- A_WT_ where A_MT_ was the area of the kinetic curve of the mutant and A_WT_ was the area of the kinetic curve of the wild type detected in the same well. Relevant difference were stated when ΔA was higher than +20000 (phenotype gained by the mutant) or lower than -20000 (phenotype lost by the mutant).

### Ethics

The studies reported in the present work do not present ethics concerns.

## Results and Discussion

Resistance to biocides is frequently due to the overexpression of efflux pumps [15–19]. This overexpression can be transient if the biocide is an effector of the transcriptional regulator of the efflux pump, or constitutive if the biocide selects mutants that overexpress the efflux pump [15]. We had previously shown that triclosan induces the expression of the *S*. *maltophilia* efflux pump SmeDEF through its binding to SmeT [28], the transcriptional repressor of the system [29,30]. This biocide-mediated transient expression of the pump implies the extrusion of triclosan as well as the efflux of several antibiotics, including quinolones, erythromycin, tetracycline and chloramphenicol that are substrates of SmeDEF [46], which means that acquisition of biocide resistance mediated by transient overexpression of SmeDEF also confers low-level antibiotic resistance.

To analyze whether or not other commonly used biocides can also select for antibiotic resistance in *S*. *maltophilia*, we analyzed two of such biocides, benzalkonium chloride and hexachlorophene, and studied both transient induction of resistance and selection of resistant mutants.

### Hexachlorophene and benzalkonium chloride bind Smet

**Fig 1:**
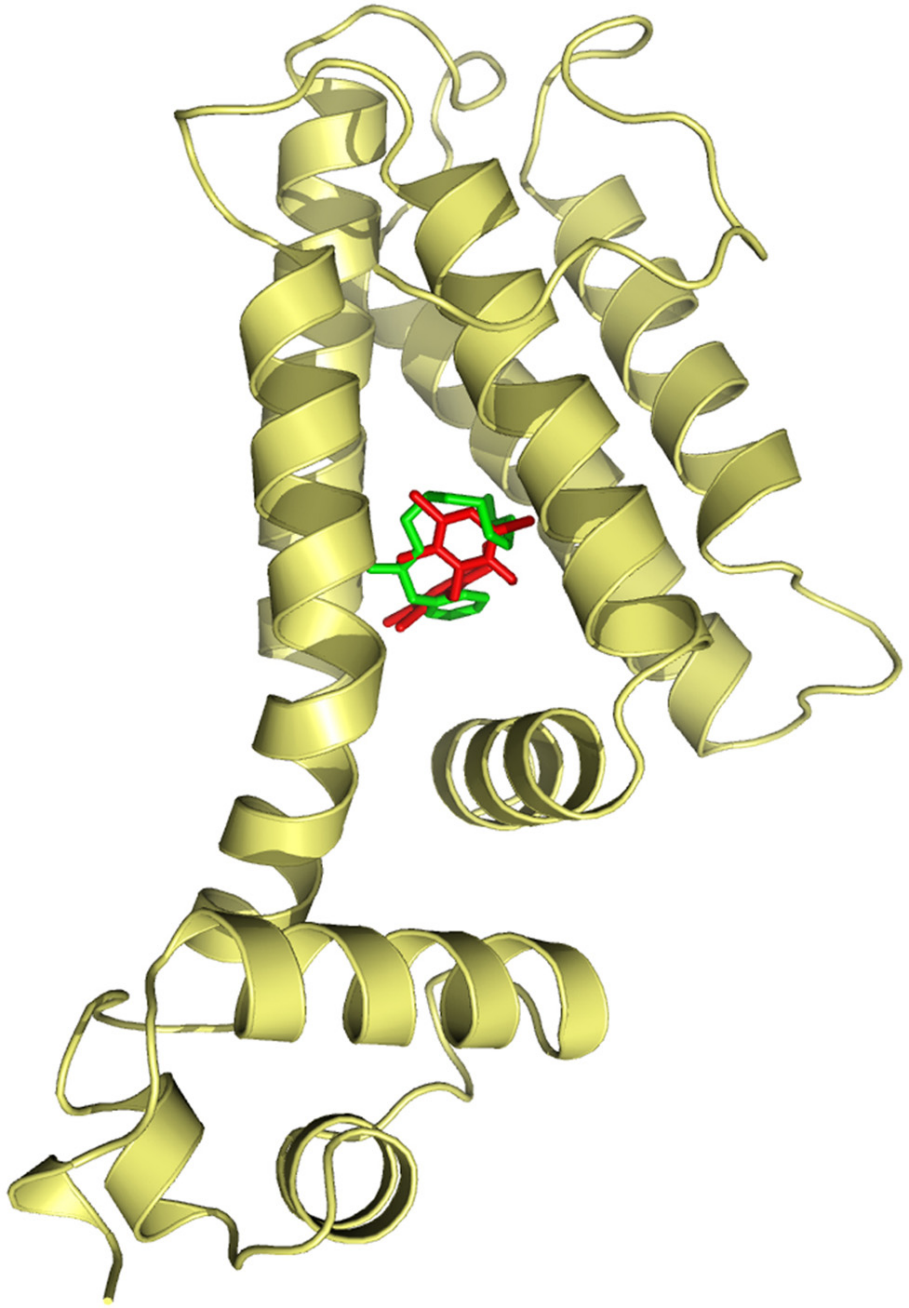
Docking of biocides to SmeT. The figure shows the best binding conformation of hexachlorophene (red) and benzalkonium (green) to the pocket of the protein. For both biocides the complex effector-protein was stabilized by hydrogen bonds and principally by hydrophobic interactions.

The expression of *smeDEF* is regulated by SmeT[30], a repressor located upstream the operon encoding this efflux pump [30]. We have previously shown that triclosan [28], as well as several different plant-produced flavonoids [36] can bind SmeT. As the consequence of such binding, in the presence of these effectors, SmeT is released from its operator sequence and SmeDEF is overexpressed. To address whether or not benzalkonium chloride or hexachlorophene might also be effectors of SmeT, we predicted the ability of SmeT to accommodate in its binding pocket these two biocides. For this purpose, we preformed a modeling approach using the information derived from the crystal structure of SmeT in the presence and in the absence of triclosan [28,29]. The results are shown in Fig 1 and predict that SmeT can indeed bind both hexachlorophene and benzalkonium chloride.

**Fig 2:**
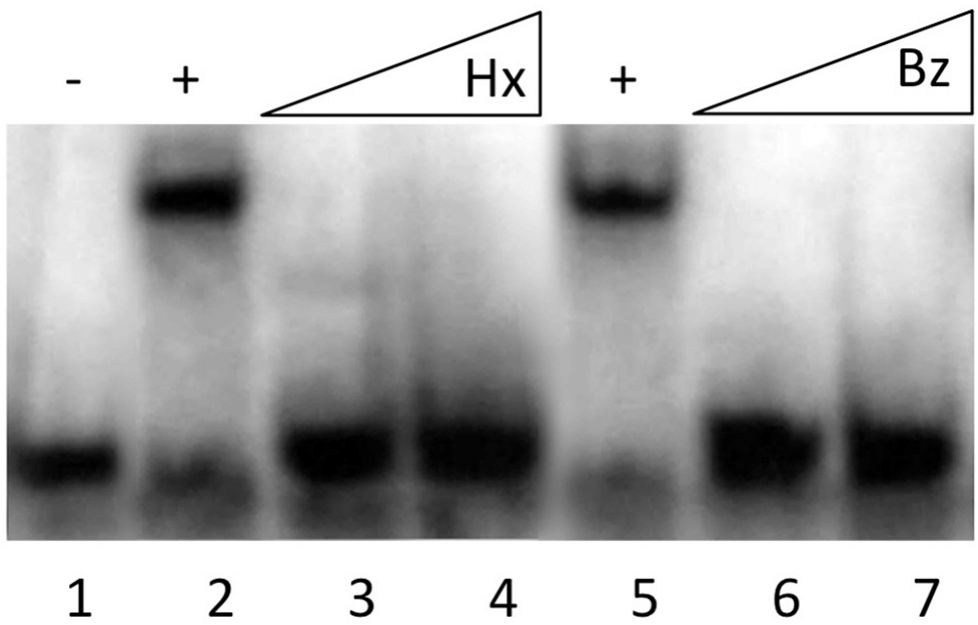
Effect of hexachlorophene and benzalkonium chloride on the capability of SmeT to bind its operator DNA. A γ-[^32^P] labeled double stranded DNA fragment containing the SmeT operator site (lane 1) was incubated with SmeT (0.2 μM) for 20 min at room temperature (lane 2). Subsequently, increasing concentrations of benzalkonium chloride (Bz) or hexachlorophene (Hx) were added (0.1 mM and 0.2 mM of each). As shown both biocides break the SmeT-DNA complex.

To validate these predictions, we studied the effect of these two biocides on the capability of SmeT to bind its operator. For this purpose, we performed EMSA assays using purified SmeT and the intergenic *smeT-smeDEF* region, which contains the operator regions of this regulator, in the presence and in the absence of the biocides. As shown in Fig 2, both biocides impede the binding of SmeT to its operator. This result confirms the docking predictions and shows that benzalkonium chloride and hexachlorophene can bind SmeT, releasing this repressor from its cognate operator DNA.

### Benzalkonium chloride triggers the expression of *smeDEF* without producing relevant changes in the susceptibility of *S*. *maltophilia* to antibiotics.

**Fig 3:**
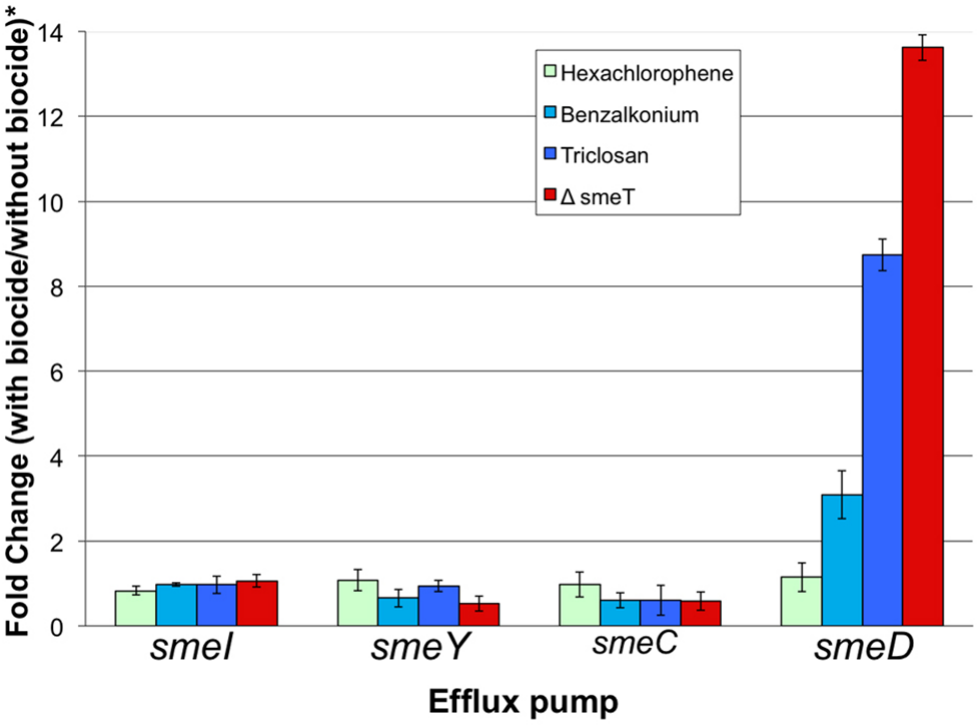
Effect of biocides on the expression of *S*. *maltophilia* efflux pumps. The amount of mRNA of efflux pumps in the presence of biocides was measured by real time RT-PCR. Fold changes were estimated with respect to the value determined for the wild type strain grown in the absence of any biocide. *In the strain D457R, expression of *smeDEF* is fully derepressed, in the absence of the biocide, due to a mutation that inactivates SmeT. As shown, benzalkonium chloride can induce expression of *smeD*, although the level reached is lower than in the D457R mutant, whereas the expression of the other analyzed efflux pumps is not induced by the biocide.

To ascertain whether the capability of binding to SmeT correlates with *smeDEF* induction, expression of *smeD* was measured in the presence and in the absence of the biocides. Expression of other *S*. *maltophilia* relevant efflux pumps, namely *smeABC*, *smeIJK* and *smeYZ* [47–49] was also measured. As shown in Fig 3, in agreement with the results from the EMSA assay, benzalkonium chloride triggers *smeD* expression. Nevertheless, it does not trigger the expression of the other tested efflux pumps. In addition, we could not detect any induction in the case of hexachlorophene.

Since *smeDEF* is overexpressed in the presence of benzalkonium chloride, an increase in the MICs of those antibiotics that are extruded by SmeDEF can be expected when *S*. *maltophilia* grows in the presence of subinhibitory concentrations of such biocide. To address this possibility, synergy assays were performed by crossing a ciprofloxacin E-test strip with a strip containing benzalkonium chloride. As shown in S1A Fig, Supporting information, in the presence of the biocide, ciprofloxacin MICs increased from 0,75 μg/ml to 1,5 μg/ml, an increase much lower than that observed in the mutant D457R, which constitutively overexpresses SmeDEF [46]. To further confirm this result, the effect of biocides (at the concentrations that induce *smeD* expression) on the growth of *S*. *maltophilia* in the presence and in the absence of ciprofloxacin was measured. As shown in S1D Fig, Supporting information, the presence of benzalkonium chloride renders a little improvement in the growth of *S*. *maltophilia* in the presence of ciprofloxacin. The concentrations of the biocide required for such effect impaired *S*. *maltophilia* growth in the absence of quinolones indicating that the range of benzalkonium chloride concentrations at which transient resistance might be induced are close to the inhibitory biocide concentrations. Consistent with the synergy assay performed with the E-test strips, we did not detect any reduction in the susceptibility to ciprofloxacin of *S*. *maltophilia* when hexachlorophene was present.

### Selection of *S*. *maltophilia* biocide resistant mutants

The possibility of selecting resistant mutants depends on two different parameters; one is the mutation rate leading to resistance [50], which measures the probability of emergence of mutants; second is the fitness cost, which is highly relevant for the fixation of the mutation in the bacterial population [51–53]. The mutation rates of resistance to triclosan, benzalkonium chloride and hexachlorophene were measured and resulted to be respectively 7.3 E^-7^ ± 1.0 E^-7^, 3.3 E^-8^ ± 8.8 E^-9^ and 5.6 E^-11^ ± 2.6 E^-11^. These data indicate that the probability of selecting biocide resistant mutants is higher for triclosan, followed by benzalkonium chloride, whereas the determined mutation rate for hexachlorophene is below the normal *S*. *maltophilia* mutation rate [54]. These results likely fit with the fact that triclosan has a single, well defined target [55], whereas benzalkonium chloride presents multiple targets, including the cell membrane, and hexachlorophene is a membrane-damaging biocide.

### *S*. *maltophilia* benzalkonium chloride resistant mutants may present reduced susceptibility to antibiotics

We have previously shown that triclosan, an effector of the repressor of SmeDEF SmeT [28], selects resistant mutants that overproduce SmeDEF and consequently are resistant to antibiotics [15]. Since both benzalkonium chloride and hexachlorophene bind SmeT, it is conceivable to think that these biocides can select antibiotic resistant mutants overexpressing SmeDEF. To analyze this possibility, the MICs of different antibiotics were determined in a set of selected benzalkonium chloride and hexachlorophene resistant mutants. Two of seven mutants selected in the presence of benzalkonium chloride namely Bz4 and Bz6 (benzalkonium chloride MIC for the mutants 256 μg/ml and for the wild-type strain 128 μg/ml), presented reduced susceptibilities to different antibiotics (Table 1) and were chosen for further studies. On the contrary, none of the studied hexachlorophene resistant mutants (hexachlorophene MIC for the mutants 32 μg/ml and for the wild-type strain 8 μg/ml), presented changes in their susceptibility to antibiotics.

**Table 1:** Susceptibility to antibiotics of *S*. *maltophilia* benzalkoniumbenzalkonium chloride resistant mutants, and their complemented derivatives.

MIC (mg/L)	Strains									
	D457 (wt)				Bz4			Bz6		
Plasmid		pVLT31	pBS37	pBS41		pVLT31	pBS42		pVLT31	pBS38
**Amikacin**	8	4	3	4	8	6	8	16	16	16
**Gentamicin**	2	2	2	2	2	2	2	2	3	3
**Aztreonam**	24	12	16	12	>256	>256	>256	128	48	48
**Cefepime**	16	12	16	12	48	48	48	24	24	24
**Ceftazidime**	0.38	0.25	0.5	0.38	1.5	1	1.5	1	1.5	1.5
**Piperacillin**	64	48	64	48	>256	>256	>256	>256	>256	>256
**Polymyxin B**	6	6	6	6	12	6	6	12	6	6
**Chloramphenicol**	6	8	6	6	6	6	8	6	12	12
**Gatifloxacin**	0.125	0.125	0.190	0.125	0.38	0.38	0.38	2	1.5	1.5
**Levofloxacin**	0.38	0.25	0.38	0.25	0.5	0.5	0.5	2	1.5	1.5
**Ofloxacin**	0.75	0.75	0.75	0.75	1.5	1.5	1.5	8	8	6
**Ciprofloxacin**	1	1.5	1.5	1	2	2	2	>32	>32	>32

Overexpression of SmeDEF is associated with increased MICs of quinolones and chloramphenicol, among other antibiotics, without changes in the susceptibility to beta-lactams [39,46]. As shown in Table 1 the phenotype of the studied benzalkonium chloride resistant mutants is not compatible with this phenotype, suggesting that SmeDEF is not overexpressed in these mutants. To address which is could be the cause of resistance, we sequenced the genomes of these two mutants. In both cases, the sequences of the mutants did not present any insertion or deletion and single SNPs were found for each of the mutants. Bz4 displayed a mutation (Glu112Stop) in a gene encoding a hypothetical membrane transporter. Bz6 presented a mutation (Arg81Ser) in the gene encoding PhoP, the DNA-binding response regulator of the two-component regulatory system PhoPQ [56]. To ensure the presence of these SNPs in the genome of the mutants, the regions comprising the mutations were amplified by PCR and sequenced in the mutants and in their parental wild-type strain D457. In both cases, the identity of the SNPs conferring resistance to biocides and antibiotics were confirmed in the mutants, whilst they were not present in the genome of the parental wild-type strain.

To further analyze the contribution of these mutations in the phenotype, the Bz4 and Bz6 mutants were complemented with the wild-type alleles of SMD_2876 and *phoP* respectively, as described in experimental procedure. The overexpression of wild-type genes in the mutants, Bz4 and Bz6, had no effect on the susceptibility to antibiotics (Table 1). However when *S*. *maltophilia* D457 was complemented with the mutated alleles of SMD_2876 and *phoP*, the overexpression of mutated genes had some effect, mainly the overexpression of mutated *phoP* (pBS37), producing a small decrease in the susceptibility to some antibiotics as ceftazidime of gatifloxacine (Table 1). To further analyze this partial complementation, spot tests were performed as described in Methods. As shown in S2 Fig, Supporting information, the expression of the mutant allele of *phoP* (pBS37) slightly reduces the susceptibility of the wild-type strain D457 to ciprofloxacin and ceftazidime, whereas the introduction of the mutant allele of SMD_2876 slightly reduces the susceptibility to ceftazidime, without a clear effect on ciprofloxacin.

Although the genomes of the mutants were sequenced with a 100% coverage, with an average coverage of 75x, and the only detected SNPs were those described above, we did not observe complementation upon expression of the wild-type allele of the corresponding genes in the mutant strains, and a very minor complementation of the phenotype when the mutant allele was expressed in the wild-type strain. There are different explanations for these observations. First, the observed mutations do not inactivate the corresponding proteins; the complemented strains are hence merodiploids, which phenotypes are difficult to predict. Second, there are some mutations that cannot be complemented by classical tests in which the normal allele is introduced. As an example, both the absence and the increased expression of the cyanide-insensitive CIO terminal oxidase make *Pseudomonas* more susceptible to antibiotics [57]. Third, even though our whole-genome sequence results are robust and the possibility of two mutations in a single step mutant (as those selected in our work) is below 10^−16^, taking into consideration already described *S*. *maltophilia* mutation rates [54], this possibility still remains. As a conclusion, although genomic information and partial complementation suggest that mutations at *phoP* and at SMD_2876 are responsible of the observed phenotype, we cannot provide a robust functional validation supporting this suggestion, and more work will be needed to determine the role of these two genes in benzalkonium chloride resistance in *S*. *maltophilia*.

### Fitness costs associated to the acquisition of biocide resistance by *S*. *maltophilia*

**Fig 4:**
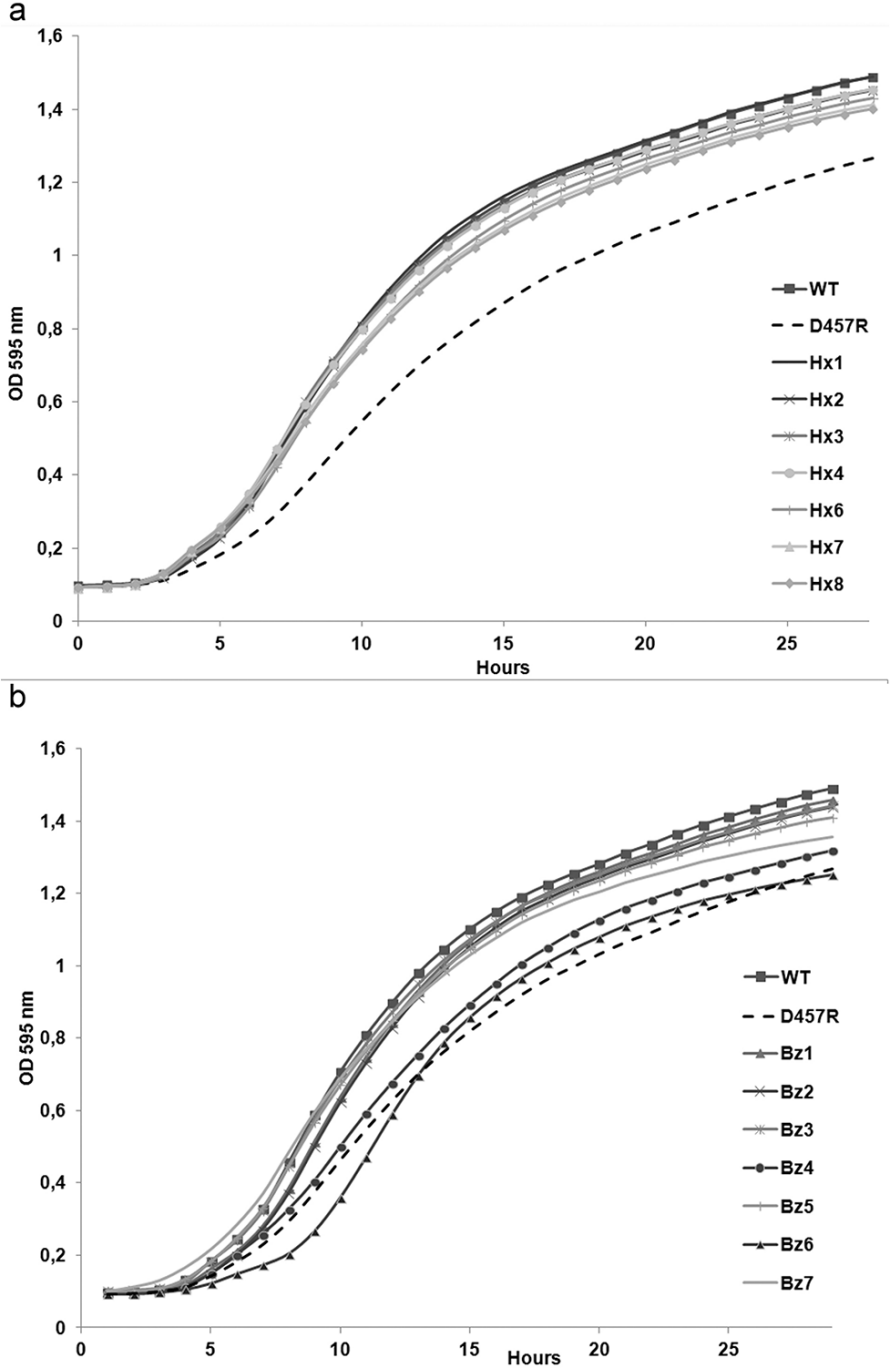
Fitness costs associated to biocide resistance in *S*. *maltophilia*. The growth of the different mutants was compared with the wild-type strain and with D457R, a triclosan resistant mutant, which overexpresses SmeDEF and is impaired in growth. As shown, resistance to hexachlorophene (a) is not associated with relevant fitness costs. Benzalkonium chloride resistant mutants present different fitness (b); the mutants Bz4 and Bz6, which present reduced susceptibility to different antibiotics, are impaired in their growth as compared with their parental wild-type strain D457.

**Fig 5:**
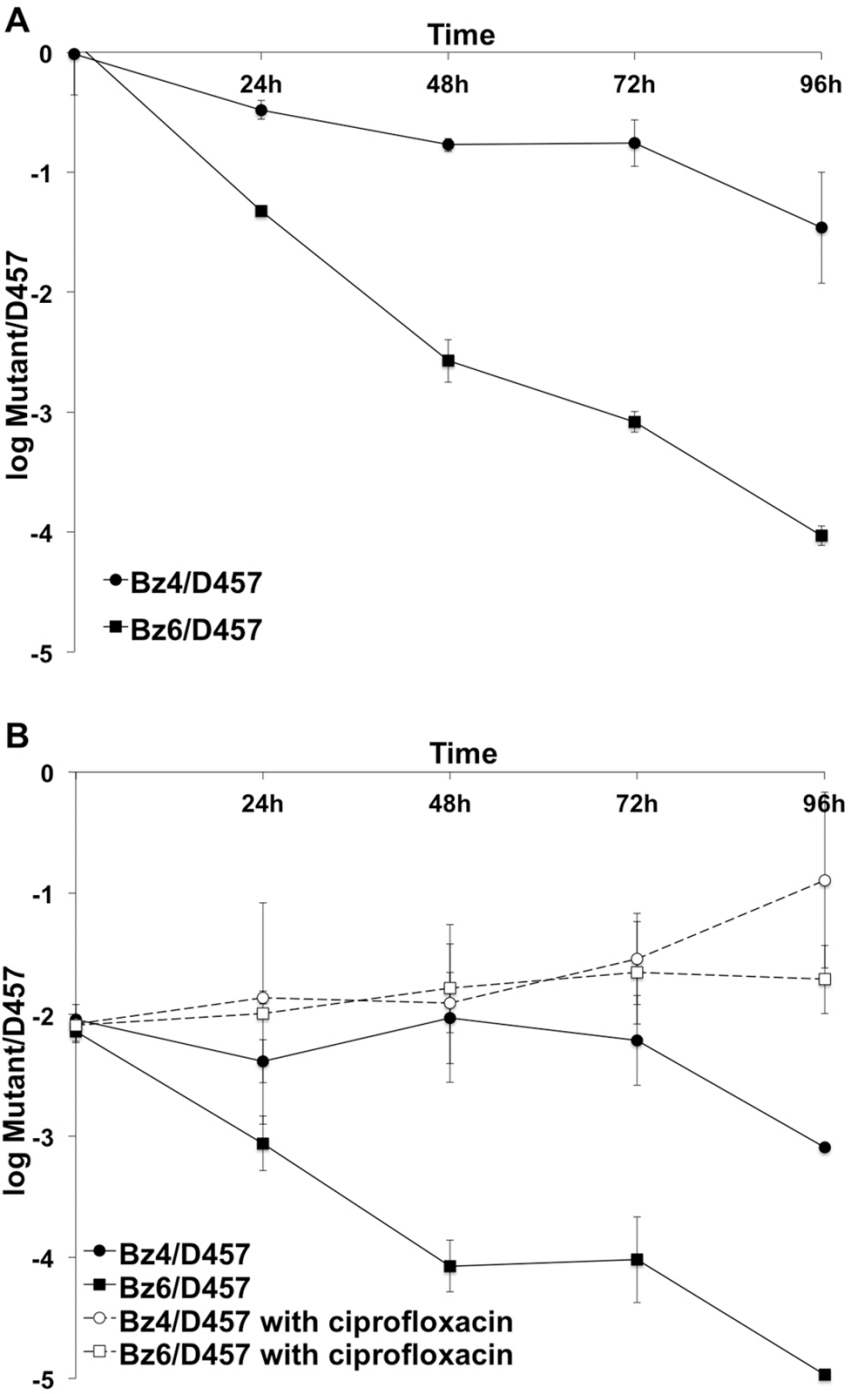
*S*. *maltophilia* D457 and benzalkonium chloride resistant mutants, Bz4 and Bz6 competition assays. *S*. *maltophilia* D457 displace the mutants Bz4 and Bz6, while mutants Bz4 and Bz6 displace *S*. *maltophilia* D457 in presence of antibiotic. The presence of low concentration of ciprofloxacin (0.5 μg/ml) makes disappear fitness cost associated to mutations. A, same number of cfu (50%) of *S*. *maltophilia* D457 and mutant was used to inoculated. B, 99% of *S*. *maltophilia* D457 and 1% of mutant was used to inoculate. Full circle, competition of Bz4 with D457; full square, competition of Bz6 with D457; empty circle, competition of Bz4 with D457 and 0.5 μg/ml ciprofloxacin; empty square, competition of Bz6 with D457 and 0.5 μg/ml ciprofloxacin.

As above stated, the fixation of a given mutation is a function of the associated fitness costs; mutants presenting high fitness costs will be outcompeted by wild-type strains in the absence of selection, whereas the chances for spread and fixation are higher for those mutants presenting very low (if any) fitness cost [51–53]. To address the effect of biocide resistance mutations on *S*. *maltophilia* fitness, we compared the growth of the different mutants to that of the wild-type strain alone and in co-cultures. We have already shown that triclosan selects mutants that overexpress SmeDEF [15] and that overexpression of this efflux pump is associated with a relevant fitness cost [58]; consequently, the SmeDEF hyperexpressing mutant D457R [32] was included in the study. As shown in Fig 4, only the two mutants displaying a reduced susceptibility to antibiotics, Bz4 and Bz6, presented a lower growth rate than the wild-type parental strain. To further study the effect of these mutations on *S*. *maltophilia* fitness, competition experiments were performed in the absence and in the presence of antibiotics. For the second situation the wild-type strain was given an initial advantage (99% wild-type vs 1% mutant strain). In agreement with our previous results, the benzalkonium chloride resistant mutants (Bz4 and Bz6) were displaced by wild-type strain D457 when growing in co-culture (Fig 5). Nevertheless, the addition of sub-MIC antibiotic concentrations increased the fitness of the mutant strains, which are not outcompeted by the wild-type strain. This result is in agreement with previous findings showing that sub-MIC antibiotic concentrations can select antibiotic resistant mutants [59].

This suggests that antibiotic resistant mutants selected in the presence of benzalkonium chloride might be compromised for their spread and hence for their fixation in the population, in absence of antibiotic, whereas the presence of antibiotics, even at sub-MIC concentrations, may favor their spread. Concerning hexachlorophene mutants, none of them presented an impaired growth rate as compared with the wild-type strain (Fig 4). However, since none of them presented a reduced susceptibility to antibiotics, the risk that hexachlorophene selects antibiotic resistant mutants is low.

In addition of producing a non-specific metabolic burden, which is reflected in a reduction on the growth rate, acquisition of resistance may produce specific changes in the bacterial metabolism. To study this possibility the use of nutrients of the Bz4 and Bz6 mutants was compared with that of the wild-type strain using BIOLOG phenotypic microplates. Quite surprisingly, the most noticeable effect observed for both mutants was an increased capability of using dipeptides and tripeptides as nitrogen sources an issue that does not explain the observed growth defect in LB (Table 2). This result [58] suggests that in an environment containing dipeptides, the studied benzalkonium chloride resistant mutants might not have relevant fitness costs.

**Table 2:** Phenotypes gained and lost by the mutant strains D457R, Bz4 and Bz6, in comparison with their parental wild-type strain as measured using PM panels PM1-PM8.

MUTANT	PM	WELL	ASSAY	COMPOUND	∆A*
**D457R**	*PM4*	*B9*	*P-source utilization*	*GUANOSINE-3'-MONOPHOSPHATE*	*-22353*
	*PM4*	*D9*	*P-source utilization*	*URIDINE-3'-MONOPHOSPHATE*	*-47968*
	PM6	H1	N-source utilization	ILE-TRP	25752
	PM7	C12	N-source utilization	PHE-PHE	20318
	PM8	H8	N-source utilization	GLY-PHE-PHE	32344
**Bz4**	PM1	D1	C-source utilization	L-ASPARAGINE	22085
	PM3	A12	N-source utilization	L-GLUTAMNIC ACID	21345
	PM3	G12	N-source utilization	ALFA-AMINO-N-VALERIC ACID	21861
	PM6	H1	N-source utilization	ILE-TRP	20091
	PM7	A12	N-source utilization	LYS-PHE	23158
	PM7	C12	N-source utilization	PHE-PHE	26361
	PM7	D3	N-source utilization	PHE-TRP	21355
	PM8	H6	N-source utilization	GLY-GLY-PHE	26718
	PM8	H8	N-source utilization	GLY-PHE-PHE	33835
**Bz6**	PM1	F1	C-source utilization	GLYCYL-L-ASPARTIC ACID	22441
	PM1	G1	C-source utilization	GLYCYL-L-GLUTAMNIC ACID	20444
	PM3	G12	N-source utilization	ALFA-AMINO-N-VALERIC ACID	29933
	*PM4*	*D10*	*P-source utilization*	*URIDINE-5'-MONOPHOSPHATE*	*-49500*
	*PM4*	*F3*	*S-source utilization*	*THIOSULPHATE*	*-21543*
	*PM4*	*G1*	*S-source utilization*	*N-ACETYL-L-CYSTEINE*	*-24720*
	*PM4*	*H1*	*S-source utilization*	*L-DJENKOLIC ACID*	*-26776*
	PM6	B10	N-source utilization	ARG-ILE	20636
	PM6	D12	N-source utilization	GLU-TYR	30006
	PM6	G1	N-source utilization	HIS-TYR	34606
	PM6	G8	N-source utilization	ILE-ILE	26385
	PM6	G10	N-source utilization	ILE-PHE	28221
	PM6	H1	N-source utilization	ILE-TRP	35899
	PM7	C11	N-source utilization	PHE-ILE	28339
	PM7	C12	N-source utilization	PHE-PHE	22980
	PM7	D12	N-source utilization	PRO-TYR	33964
	PM7	E9	N-source utilization	SER-TYR	26163
	PM7	G6	N-source utilization	TYR-GLN	31486
	PM7	G7	N-source utilization	TYR-GLU	33400
	PM7	G9	N-source utilization	TYR-HIS	27548
	PM7	G10	N-source utilization	TYR-LEU	20293
	PM7	H10	N-source utilization	VAL-TYR	29621
	PM8	E1	N-source utilization	THR-PHE	20391
	PM8	E11	N-source utilization	VAL-PHE	24611
	PM8	H7	N-source utilization	VAL-TYR-VAL	42095
	PM8	H8	N-source utilization	GLY-PHE-PHE	32772
	PM8	H12	N-source utilization	TRY-GLY-GLY	22469

* ΔA was defined as ΔA = AMT- AWT where AMT was the area of the kinetic curve of the mutant and AWT was the area of the kinetic curve of the wild type parental strain D457 detected in the same well. Relevant difference were stated when ΔA was higher than +20000 (phenotype gained by the mutant) or lower than -20000 (phenotype lost by the mutant, italics in the table)

## Concluding Remarks

By using *in silico* and biochemical approaches, it can be predicted that the biocides hexachlorophene and benzalkonium chloride should induce transient antibiotic resistance mediated by the overexpression of SmeDEF. Nevertheless, microbiological phenotypic assays showed these predictions not to be true. The presence of the studied biocides did not reduced significantly the susceptibility to antibiotics, most likely because, as previously discussed for triclosan, the concentrations required for inducing *smeDEF* expression are likely in the range of the lethal concentrations for these biocides [28].

In agreement with these results, neither the benzalkonium chloride nor the hexachlorophene resistant mutants overexpressed SmeDEF. However, whereas antibiotic resistance was not associated with resistance to hexachlorophene, some benzalkonium chloride resistant mutants presented a reduced susceptibility to different antimicrobials. Altogether our results show that both triclosan and benzalkonium chloride can select antibiotic resistant mutants, the mechanism of resistance being different in each case, whereas no antibiotic resistant mutants are selected by hexachlorophene. The fact that in all cases the acquisition of resistance impairs bacterial fitness may reduce the chances for dissemination and fixation of this type of mutants among *S*. *maltophilia* population.

## Supporting Information

S1 Fig Synergy of biocides with antibiotics. E-test ciprofloxacin (CI) strips were crossed with strips containing either ethanol (the solvent used for the biocides' dilutions) (a), benzalkonium (b) or hexachlorophene (c). CI strips without any added biocide were used as control in each experiment. As shown, a little increase in MIC was observed in the presence of benzalkonium, whereas the effect of hexachlorophene on *S*. *maltophilia* susceptibility to ciprofloxacin was undetectable under these conditions. To further analyze the effect of biocides on the susceptibility of *S*. *maltophilia* to quinolones, the effect of benzalkonium (3.5 μg/ml) (d) and hexachlorophene (1.5 μg/ml) (e) on the growth of *S*. *maltophilia* in the presence of ciprofloxacin (2 μg/ml) was measured. As shown, the presence of benzalkonium just partially retrieves the effect of ciprofloxacin on *S*. *maltophilia* growth, whereas hexachlorophene did not alter the effect of the quinolone on *S*. *maltophilia* growth. To note that the minor effect of benzalkonium on *S*. *maltophilia* susceptibility to quinolones is observed at concentrations in which the biocide impairs bacterial growth by its own (d).
(TIF)

S2 Fig Spot growth assay of complemented *S*. *maltophilia* D457, Bz4 and Bz6 mutants. Serial dilution (without dilution (WD),10^0^, 10^−1^, 10^−2^ and 10^−3^) of *S*. *maltophilia* D457, Bz4 and Bz6 mutants complemented with *phoP* and SMD_2876 wild type and mutated alleles (see experimental procedures) were spotted in plates of Mueller Hinton II agar plus 0.5mM IPTG without or with antibiotics.
(TIF)
