# Supplementary figures and images for: Predictive Studies Suggest that the Risk for the Selection of Antibiotic Resistance by Biocides Is Likely Low in Stenotrophomonas maltophilia
Source: PLoS One. 2015 Jul 22;10(7):e0132816. doi: 10.1371/journal.pone.0132816 (PMC4511778; doi:10.1371/journal.pone.0132816)

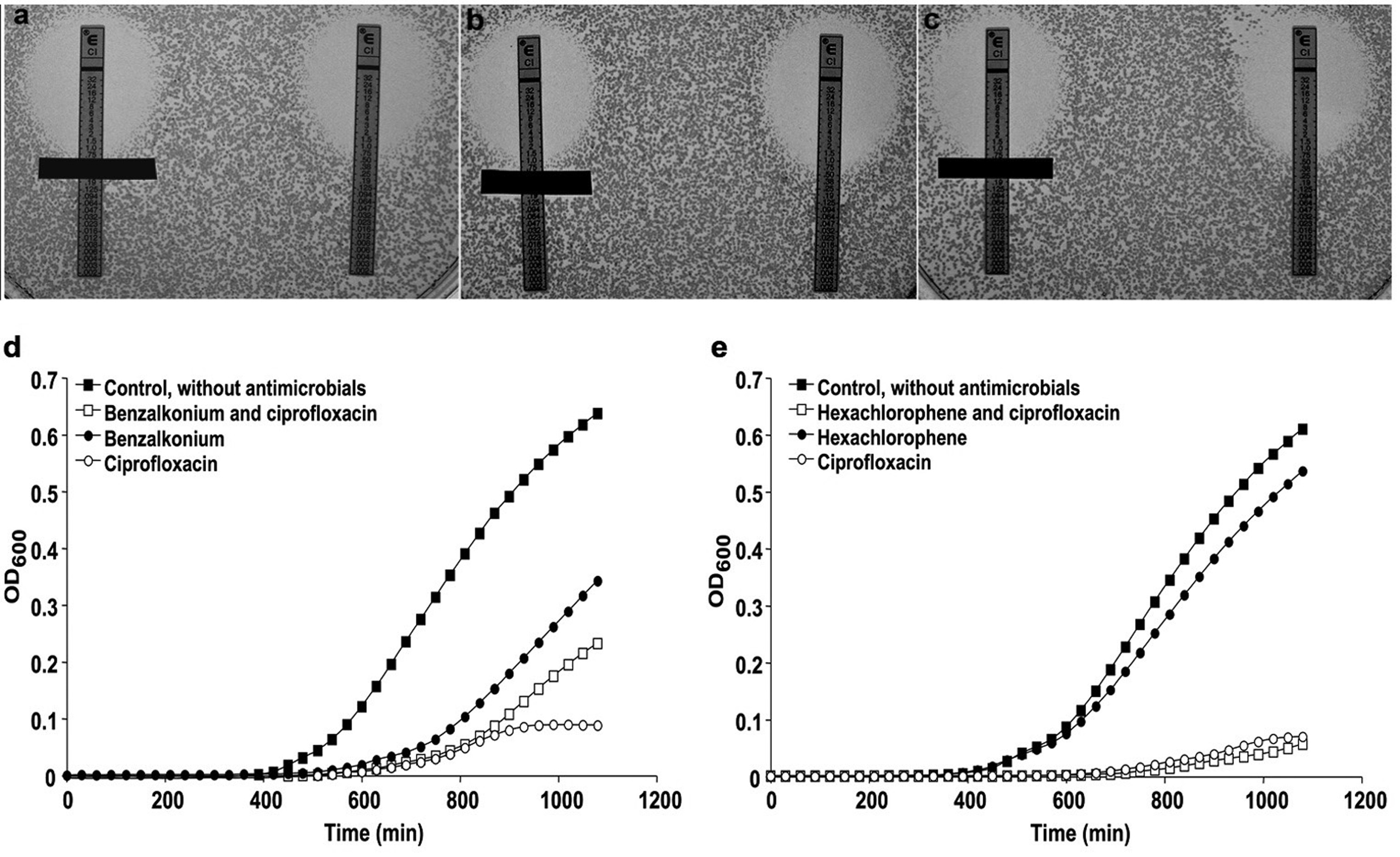

Supplement: S1 Fig — E-test ciprofloxacin (CI) strips were crossed with strips containing either ethanol (the solvent used for the biocides' dilutions) (a), benzalkonium (b) or hexachlorophene (c). CI strips without any added biocide were used as control in each experiment. As shown, a little increase in MIC was observed in the presence of benzalkonium, whereas the effect of hexachlorophene on S. maltophilia susceptibility to ciprofloxacin was undetectable under these conditions. To further analyze the effect of biocides on the susceptibility of S. maltophilia to quinolones, the effect of benzalkonium (3.5 μg/ml) (d) and hexachlorophene (1.5 μg/ml) (e) on the growth of S. maltophilia in the presence of ciprofloxacin (2 μg/ml) was measured. As shown, the presence of benzalkonium just partially retrieves the effect of ciprofloxacin on S. maltophilia growth, whereas hexachlorophene did not alter the effect of the quinolone on S. maltophilia growth. To note that the minor effect of benzalkonium on S. maltophilia susceptibility to quinolones is observed at concentrations in which the biocide impairs bacterial growth by its own (d). (TIF) [file pone.0132816.s001.tif]

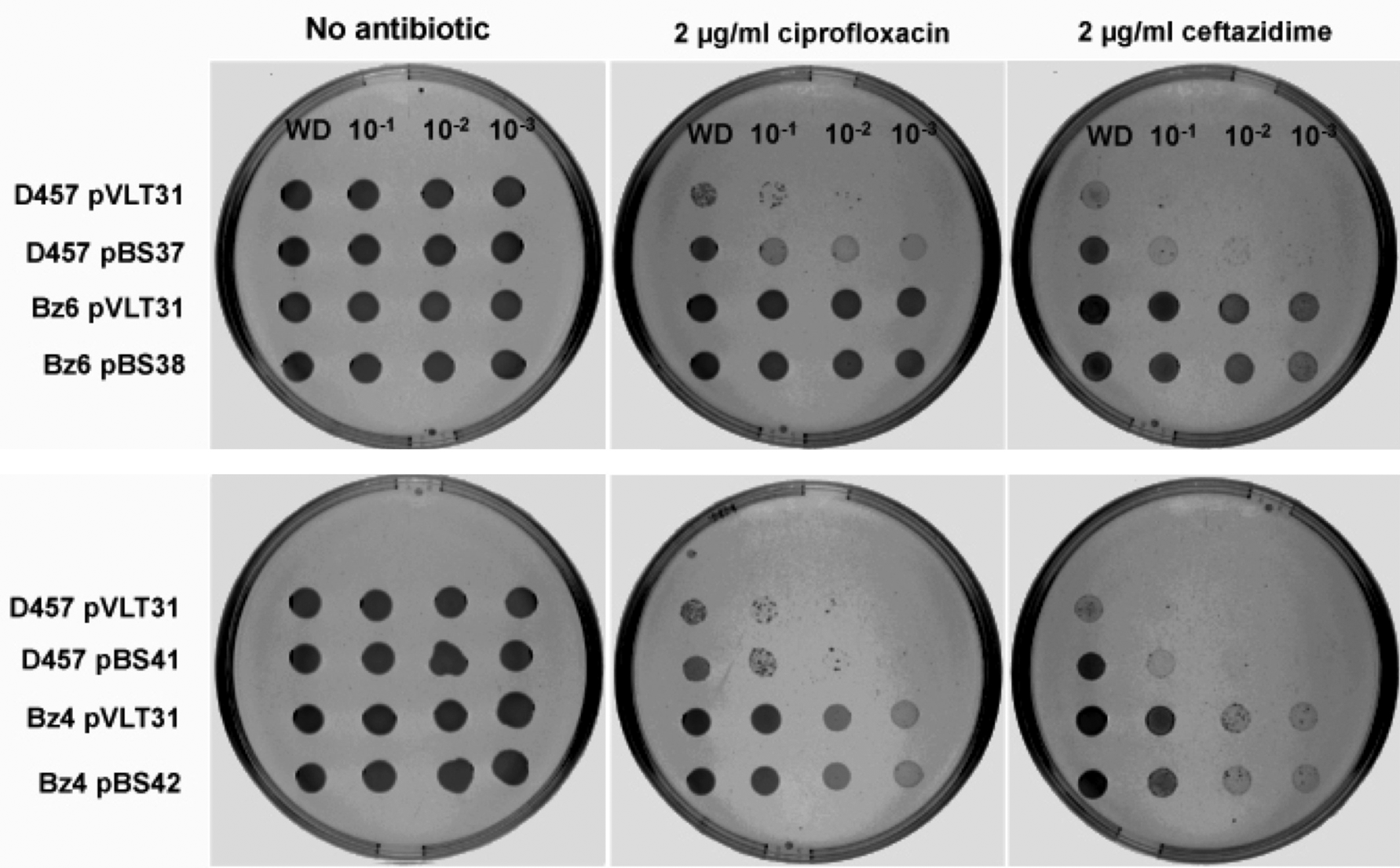

Supplement: S2 Fig — Serial dilution (without dilution (WD),100, 10−1, 10−2 and 10−3) of S. maltophilia D457, Bz4 and Bz6 mutants complemented with phoP and SMD_2876 wild type and mutated alleles (see experimental procedures) were spotted in plates of Mueller Hinton II agar plus 0.5mM IPTG without or with antibiotics. (TIF) [file pone.0132816.s002.tif]
